# Comparative analysis of lipopolysaccharides of pathogenic and intermediately pathogenic *Leptospira* species

**DOI:** 10.1186/s12866-015-0581-7

**Published:** 2015-10-30

**Authors:** Kailash P. Patra, Biswa Choudhury, Michael M. Matthias, Sheyenne Baga, Keya Bandyopadhya, Joseph M. Vinetz

**Affiliations:** Division of Infectious Diseases, Department of Medicine, Biomedical Research Facility, University of California San Diego, 9500 Gilman Drive, BRF 2, Room 5220, La Jolla, 92093-0760 California USA; Glycotechnology Core Resources, University of California San Diego, 9500 Gilman Drive, BRF 2, Room 4243, La Jolla, 92093-0687 California USA

**Keywords:** Lipopolysaccharide, *Leptospira*, Chemical composition, O-antigen diversity

## Abstract

**Background:**

Lipopolysaccharides (LPS) are complex, amphipathic biomolecules that constitute the major surface component of Gram-negative bacteria. *Leptospira*, unlike other human-pathogenic spirochetes, produce LPS, which is fundamental to the taxonomy of the genus, involved in host-adaption and also the target of diagnostic antibodies. Despite its significance, little is known of *Leptospira* LPS composition and carbohydrate structure among different serovars.

**Results:**

LPS from *Leptospira interrogans* serovar Copenhageni strain L1-130, a pathogenic species, and *L. licerasiae* serovar Varillal strain VAR 010, an intermediately pathogenic species, were studied. LPS prepared from aqueous and phenol phases were analyzed separately. *L. interrogans* serovar Copenhageni has additional sugars not found in *L. licerasiae* serovar Varillal, including fucose (2.7 %), a high amount of GlcNAc (12.3 %), and two different types of dideoxy HexNAc. SDS-PAGE indicated that *L. interrogans* serovar Copenhageni LPS had a far higher molecular weight and complexity than that of *L. licerasiae* serovar Varillal. Chemical composition showed that *L. interrogans* serovar Copenhageni LPS has an extended O-antigenic polysaccharide consisting of sugars, not present in *L. licerasiae* serovar Varillal. Arabinose, xylose, mannose, galactose and L-glycero-D-mannoheptose were detected in both the species. Fatty acid analysis by gas chromatography–mass spectrometry (GC-MS) showed the presence of hydroxypalmitate (3-OH-C16:0) only in *L. interrogans* serovar Copenhageni. Negative staining electron microscopic examination of LPS showed different filamentous morphologies in *L. interrogans* serovar Copenhageni vs. *L. licerasiae* serovar Varillal.

**Conclusions:**

This comparative biochemical analysis of pathogenic and intermediately pathogenic *Leptospira* LPS reveals important carbohydrate and lipid differences that underlie future work in understanding the mechanisms of host-adaptation, pathogenicity and vaccine development in leptospirosis.

## Background

Human leptospirosis is a common, globally-important and neglected zoonotic infectious disease, caused by spirochetes of the genus *Leptospira* [1], a disease of particular public health importance in tropical and subtropical countries [2]. Chronically infected mammalian reservoir hosts (e.g., rodents, cattle, dogs and swine) excrete the organism in urine, contaminating water and soil the principal vehicles for human infection. There are at least 22 recognized species of *Leptospira* that have been further classified into three major subgroups: Pathogenic, Intermediately Pathogenic and Non-pathogenic (saprophytes) [3], comprising ≥ 250 serotypes (“serovars”) based primarily on the immunological characterization of surface lipopolysaccharide (LPS) [4–6], a feature that distinguishes *Leptospira* from other human-infecting spirochetes, which do not produce LPS.

LPSs are complex, amphipathic biomolecules that constitute the major surface antigen of Gram-negative bacteria [7, 8]. LPS is composed of three covalently-linked distinct components: lipid A, which is the hydrophobic part embedded in the outer membrane; O-antigen or O-polysaccharide, which is extended from the cell surface to the external environment; and the core oligosaccharide, which links the O-antigen to the lipid A. LPS with and without O-antigen side chains are referred to as smooth and rough LPS, respectively. Several Gram-negative organisms have been shown to produce heterogeneous (both smooth and rough) LPS [9, 10]. *Leptospira* LPS plays an essential role in pathogenesis colonization and dissemination of *Leptospira* in mouse models [11–13]. LPS is one of the target antigen for diagnosis [14, 15] and also potential candidate for vaccine development [7, 16, 17].

In contrast to the pathogenic strain, *L. interrogans* serovar Copenhageni strain Fiocruz L1-130*,* the intermediately pathogenic strain *L. licerasiae* serovar Varillal strain VAR 010 grows rapidly *in vitro*, but neither caused disease nor was molecularly detectable in experimentally infected hamsters or mice [18]. *L. licerasiae* serovar Varillal strain VAR 010 (VAR010) causes mild disease in humans [18] compared to *L. interrogans* serovar Copenhageni strain Fiocruz L1-130 infection, which frequently result in life-threatening illness characterized by jaundice, shock, kidney injury and hemorrhage [19, 20]. These critical observations suggest that there are important biological and virulence differences between pathogenic and intermediately pathogenic *Leptospira* species, which can be further exploited by comparative analysis of key components to understand the pathogenesis and host colonization. LPS is considered as one of the central component of the pathogenesis in Gram-negative organisms including pathogenic *Leptospira species* [8, 12]. Therefore, comparative characterization of LPS of a pathogenic and intermediately pathogenic *Leptospira* species is critical to delineate sugar and fatty acid molecules essential for the pathogenesis and colonization in the host.

Apart from its overall structural similarity to Gram-negative LPS, relatively little is known about the composition and structure of *Leptospira* LPS [11, 12, 21]. Until now, the chemical composition and structure of the LPS produced by an intermediate *Leptospira* have not been studied. This is particularly important considering the reduced complexity of the *L. licerasiae* serovar Varillal rfb locus (a modest 6-gene-operon) compared to that of *L. interrogans* Copenhageni, which contains 91 genes, despite the fact that both serovars have a common reservoir host: *Rattus norvegicus* [3]. Though this association is not absolute, reservoir species are thought to maintain specific serovars such as Copenhageni in rats and Canicola in dogs. Indeed, despite differing genomic backgrounds, *L. interrogans* subtype Harjoprajitno and *L. borgpetersenii* serotype Hardjobovis are indistinguishable serologically and share a common reservoir host [22] suggesting that LPS plays an important role in determining host-specificity.

To establish the biochemical basis for the structural determination of the carbohydrate component of leptospiral LPS, the biochemical composition of LPS of the high-grade pathogenic *L. interrogans* serovar Copenhageni was compared to the LPS of the intermediately pathogenic *L. licerasiae* Varillal LPS. Further, of biological relevance given that pathogenic bacterial LPS may well be associated with host adaptation, these two *Leptospira* share *Ratttus* species as their mammalian reservoir host [18]. Importantly, the *rfb* loci of these two *Leptospira* have great differences in their complexity: *L. interrogans* serovar Copenhageni has 95 *rfb* locus genes [23], while *L. licerasiae* has 6 *rfb* locus genes [3]. This study is the first to compare the biochemical composition of LPS from pathogenic and intermediate *Leptospira* sharing a common reservoir host and having varying pathogenic potentials, and will underpin future structural studies of leptospiral LPS.

## Methods

### Ethics statement

This study was carried out in accordance with the recommendations in the Guide for the Care and Use of Laboratory Animals of the National Institutes of Health in AAALAC-approved facilities. The experimental animal work was approved by the Institutional Animal Care and Use Committee of the University of California San Diego under protocol S03128H.

### Strains and culture condition and LPS extraction

*Leptospira interrogans* serovar Copenhageni strain Fiocruz L1-130, a pathogen, was maintained *in-vitro*, passaged through hamsters, and re-isolated from infected hamster liver; low-passage derivatives (P10 or less) were used for all the experiments. The intermediate pathogen, *L. licerasiae* serovar Varillal (VAR010) was originally isolated from *Rattus* spp. in the Peruvian Amazon [18], adapted to *in-vitro* culture conditions in our laboratory, and analyzed at the whole genome level [3]. Both strains were cultivated in Ellinghausen-McCullough-Johnson-Harris (EMJH) medium (Becton Dickinson, Sparks, Maryland) with slow rotary shaking (100 rpm) at 30 °C. *Leptospira* cells were collected from mid-log cultures by centrifugation at 14,000 rpm for 30 minutes, and then washed three times with sterile PBS before LPS extraction.

Crude LPS was prepared by hot phenol-water extraction as described previously [24], and extensively dialyzed against Milli-Q water (8 changes for 4 days) to remove phenol then lyophilized. Lyophilized crude material was re-suspended in 10 mL of Mill-Q water, treated with DNase and RNase (Benzonase Nuclease, Sigma-Aldrich, St. Louis, MO) followed by proteinase-K digestion (Sigma-Aldrich, Product No. P6556). Purified LPS was pelleted by ultracentrifugation (120,000 *g* for 4 h at 4 °C), dissolved in Milli-Q water and lyophilized. Stock solutions of 1 mg/mL of purified LPS were prepared from both aqueous and phenol phases, and known amounts (by weight) were used for SDS-PAGE and chemical composition analysis.

### Chemical composition analysis of LPS extracted from aqueous (AQ) and phenol (PH) phases

Neutral monosaccharides were identified by GC-MS as their alditol acetate derivative (AA). Briefly, a known amount of LPS was spiked with 2 *μ*g of myo-inositol as internal standard, followed by hydrolysis with 2 N trifluoroacetic acid (TFA) at 100 °C for 4 h. Hydrolyzed samples were cooled to room temperature, and a dry nitrogen flush used to remove excess acid followed by repeated co-evaporation of the reaction mixture with 100 *μ*l of 50 % aqueous isopropyl alcohol. The liberated monosaccharide aldoses were further reduced to corresponding alditols by sodium borohydride solution (1 mg/mL in 1 M ammonium hydroxide) at room temperature overnight. Excess sodium borohydride was neutralized using cold aqueous 30 % acetic acid (HOAc), followed by removal of boric acid from the reaction mixture as volatile methyl borate using a dry nitrogen flush. Finally, sugar alditols were reacted with a 1:1 (v/v) mixture of pyridine and acetic anhydride at 100 °C for 1 h to form corresponding alditol acetate derivatives. After removal of excess reagent by dry a nitrogen flush, dried samples were extracted with dichloromethane and analyzed by GC-MS (Agilent Technologies 7820A GC system; 5975 Series MSD) using a Restek-5 ms (Restek Cat No. 13423) capillary column. Ultrapure helium was used as carrier gas at a linear flow rate of 1.25 mL/min and a temperature gradient (120 °C-10 °C for 1 min, 140 °C-2 °C for 1 min, 220 °C for 2 min, 5 °C for 1 min and 230 °C for 4 min). The injector temperature and transfer temperatures were maintained at 220 °C and 280 °C respectively. The constituent monosaccharides in LPS were identified and quantified by comparing the retention time and mass fragmentation pattern in electron impact (EI) positive ion mode with standard sugars.

### Fatty acid analysis of AQ and PH layers by GC-MS as fatty acid methyl ester (FAME) and trimethyl silyl ether (Tri-Sil)-FAME of hydroxyl fatty acids

Dried LPS samples (50 *μ*g) from both aqueous and phenol layers were treated with 200 *μ*L 1 M methanolic HCl prepared using a 3 N methanolic HCl kit (Supelco, Cat No. 3–3355) for 16 h at 80 °C. The samples were placed in an ice bath and slowly evaporated under a dry nitrogen stream until almost half of the sample volume had evaporated. To this reaction mixture, cold half-saturated NaCl was added followed by the addition of 1 mL chloroform. The samples were vortexed at high speed for 1 min with a 20-sec pulse, and then allowed to settle for 1 min, and then centrifuged at 2000 rpm for 2 min (Beckman Coulter, Allegra 25R centrifuge) to separate organic and aqueous layers. The lower organic layer was carefully removed to a glass hydrolyzing tube, and the aqueous layer was extracted twice with 0.5 mL of chloroform. Chloroform fractions were pooled then washed with 2.0 mL of cold Milli-Q water twice, and the organic layer was dried down. For detection of hydroxy fatty acids, the dried fatty acid methyl esters were treated with 100 *μ*L of Tri-Sil HTP reagent (Thermo Scientific, Cat No. TS-48999) at 80 °C for 30 min. The samples were cooled in an ice bath and then dried using a dry nitrogen stream, and then dissolved in hexane and analyzed by GC-MS.

FAME derivatized samples were analyzed by GC-MS (Agilent Technologies 7820A GC system; 5975 Series MSD) using a Restek-5 ms (Restek Cat No. 13423) capillary column. Ultrapure helium was used as a carrier gas at a linear flow rate of 1.1971 mL/min and the following temperature gradient (100 °C-5 °C for 1 min, 120 °C for 1 min, 3 °C for 1 min and −230 °C for 4 min). The injector temperature and transfer line temperature were maintained at 220 °C and 280 °C. Fatty acids were identified by their characteristic EI fragmentation pattern and corresponding retention times and the percentage composition calculated from the area under the respective peaks.

### KDO (2-keto-3-deoxyoctonate) analysis

KDO was analyzed using reverse phase HPLC with a C18 column after tagging with a fluorophore 4, 5 methylenedioxy-1, 2-phenylenediamine dihydrochloride (DMB) (Sigma, A89804). Briefly, 25 *μ*g of LPS was hydrolyzed using 100 *μ*L of 2 M HOAc at 80 °C for 3 h followed by removal of HOAc in a speed vac. The sample was then spin filtered using a 10 K (MW cut off) spin filter (Nanosep 10 K Omega, PALL Life Sciences, product No. OD010C34). The flow-through containing free KDO was reacted with DMB reagent at 50 °C for 2.5 h; a known amount of DMB-KDO was injected into an RP-HPLC equipped with an online fluorescence detector (Dionex Ultimate 3000 UHPLC-Focused Plus). A Phenomenex C18 reverse phase column (250 mm x 4.6 mm, 5 micron particle size) was used for HPLC analysis with an isocratic solvent mixture of consisting of methanol (8 %) and acetonitrile (5 %). For fluorescence detection, the excitation and emission wavelengths were set to 373 nm and 448 nm respectively.

### Electron microscopy

A drop of LPS solution (1 mg/mL in water) was deposited on a carbon coated mesh grid (200 lines/inch) and air-dried. The sample containing mesh was negatively stained with 2 % (w/v) uranyl acetate following a published method [18]. The air-dried stained grid was examined using a Joel 1200 Ex II TEM microscope at 80 kV available at the core facility at UC San Diego (emcore.ucsd.edu).

## Results

### Electrophoretic mobilty of LPS extracted from AQ and PH phases

**Fig. 1:**
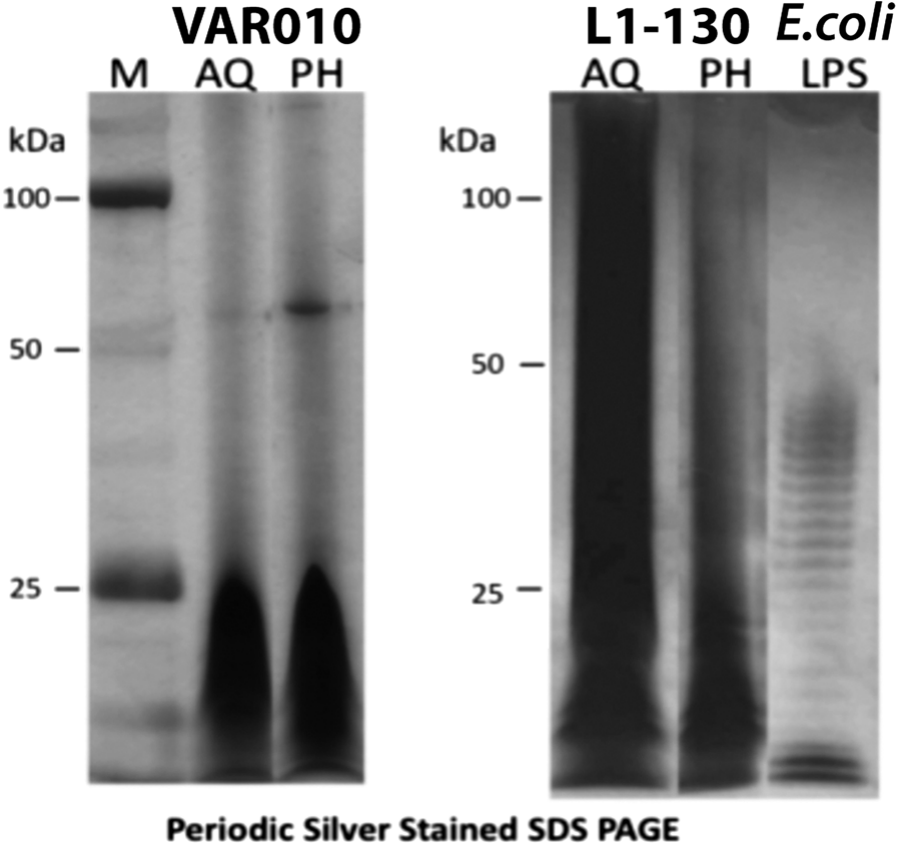
Comparative electrophoretic profile of lipopolysaccharide (LPS) extracted from pathogenic and intermediately pathogenic *Leptospira* strains. LPS was extracted from the pathogenic species, *Leptospira interrogans* serovar Copenhageni strain Fiocruz L1-130, and from the intermediately pathogenic species, *Leptospira licerasiae* serovar Varillal strain VAR010. LPS was isolated from both aqueous (AQ) and phenol (PH) layers of both species; profiles were determined by running 20 μl of LPS samples in SDS-PAGE followed by a periodic acid-silver staining method to detect LPS. *Escherichia coli* 055: B5 (*E. coli*) LPS was used as a standard LPS, and protein ladder (M) was used to localize the approximate the size of the LPS band

The silver-stained electrophoretic profiles of LPS fractions from L1-130 and VAR 10 were compared with that of an *E. coli* control (Fig. 1). The migration pattern of *Leptospira* LPS of both strains did not show the ladder-like pattern, characteristic Gram-negative smooth (S) LPS. The electrophoretic mobility of VAR010 LPS found in AQ and PH layers were similar, consisting of fast migrating low-MW band. By contrast, the AQ and PH layers of L1-130 LPS were distinct. L1-130 LPS isolated from the AQ layer displayed higher-MW to low-MW bands, forming a smear like pattern.

### GC-MS analysis of monosaccharides obtained from AQ and PH phase LPS

**Fig. 2:**
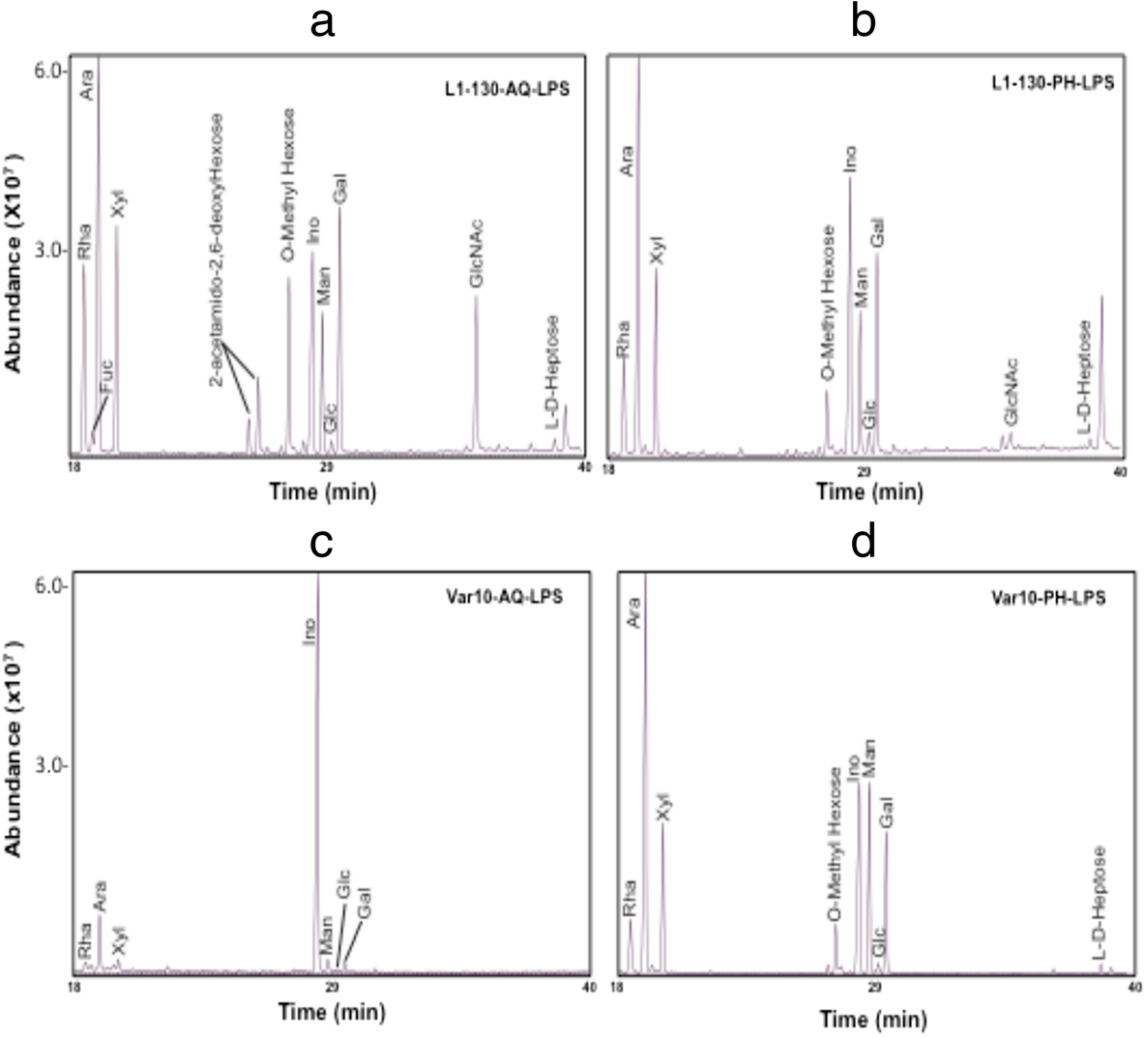
Carbohydrate composition analysis of LPS of pathogenic and intermediate pathogenic *Leptospira* strains. LPS extracted from the pathogenic species, *Leptospira interrogans* serovar Copenhageni strain Fiocruz L1-130, and from the intermediately pathogenic species, *Leptospira licerasiae* serovar Varillal strain VAR010, was subjected to gas chromatography–mass spectrometry (GC-MS) chromatography of monosaccharides as alditol acetate derivatives: (**a**) Copenhageni aqueous layer LPS; (**b**) Copenhageni phenol layer LPS; (**c**) VAR010 aqueous layer LPS; (**d**) VAR010 phenol layer LPS. Rha = 6-deoxymannopyranose; Fuc = 6-deoxygalactopyranose; Ara = arabinose; Xyl = xylose; Ino = Myo-inositol (internal standard); Man = mannose; Glc = glucose; Gal = galactose; GlcNAc = 2-N-acetylglucosamine; L-D-Heptose = L-glycero-D-mannoheptose

The carbohydrate composition of LPS of serovars Copenhageni and Varillal are summarized in Fig. 2 and Table 1. The monosaccharides present in both the strains are arabinose (Ara), rhamnose (Rha), xylose (Xyl), mannose (Man) and galactose (Gal). Several other sugar moieties are only present in the pathogen *L. interrogans* Copenhageni were detected, including fucose (Fuc, 2.7 %), N-acetylglucosamine (GlcNAc, 12.3 % in Aq-LPS and 2.03 % in Ph-LPS) and trace amounts of N-acetylgalactosamine (GalNAc, 0.35 % in the AQ layer). Two other sugars were present only in the AQ layer of serovar Copenhageni (Fig. 2), epimers of 2-N-acetyl-2, 6-dideoxy hexosamine (2,6-dideoxyHexNAc). The retention time suggests these sugars could be either gluco- or galacto- configured dideoxy HexNAc sugars, commonly known as N-acetyl quinovosamine (QuiNAc) and N-acetyl fucosamine (FucNAc). Chemical composition analysis also demonstrated the presence of O-methylated hexose residue in different fractions of LPS of both strains.

**Table 1:** Cabohydrate composition analysis of LPS of pathogenic versus intermediate pathogenic *Leptospira* strains

Sugar	Pathogenic L1-130			Intermediate pathogenic VAR010		
	AQ LPS	PH LPS	Ave (SD)	AQ LPS	PH LPS	Ave (SD)
Arabinose (Ara)	20.45	20.56	20.5 (0.05)	39.32	33.52	36.4 (5.8)
Rhamnose (Rha)	20.56	15.03	17.8 (2.8)	16.08	10.84	13.5 (2.6)
Fucose (Fuc)	2.17	0 (ND)	1.1 (1.1)	0 (ND)	0 (ND)	0 (ND)
Xylose (Xyl)	19.88	23.27	21.8 (1.7)	18.99	21.06	20.0 (1.0)
Mannose (Man)	8.74	12.73	10.7 (2.0)	15.56	19.91	17.7 (2.2)
Galactose (Gal)	14.66	16.65	15.7 (1.0)	7.87	13.2	10.5 (2.7)
Glucose (Glc)	0.89	2.29	1.59 (0.7)	2.18	1.48	1.6 (0.7)
N-acetylglucos- amine (GlcNAc)	12.3	2.03	7.2 (5.1)	0 (ND)	0 (ND)	0 (ND)
N-acetylgalacto-samine (GalNAc)	0.35	0 (ND)	0.18 (0.18)	0 (ND)	0 (ND)	0 (ND)
2-N-acetyl-2,6-dideoxy galactose (FucNAc)	+ (*)	0 (ND)	+ (*)	0 (ND)	0 (ND)	0 (ND)
2-N-acetyl-2,6-dideoxy Glucose (QuiNAc)	+ (*)	0 (ND)	+ (*)	0 (ND)	0 (ND)	0 (ND)
L-glycero-D-manno Heptose (L-D-Hep)	3.0	4.31	3.65 (0.7)	0 (ND)	4.03	2.0 (2.0)

LPS from *Leptospira interogans* serovar Copenhageni strain L1-130 (L1-130) and intermediate pathogenic, *Leptospira licerasiae* serovar Varillal strain VAR010 (VAR010) were purified from both aqueous (AQ) and phenol layer (PH) and subjected for composition analysis separately. The sugar composition values of aqueous, phenol and combined mean values (± SE) are presented as mole percentage of monosaccharides obtained in fractions. *ND* = not detected; FucNAc = 2-Nacetyl-2, 6-dideoxy Galactose and QuiNAc = 2-Nacetyl-2, 6-dideoxy Glucose (*). FucNAc and QicNAc are proposed according to the EI mass fragmentation pattern and relative retention times; however quantification could not be done due to unavailability of authentic standards for both monosaccharides

### Fatty acid composition analysis of LPS from intermediate and pathogenic strain

**Fig. 3:**
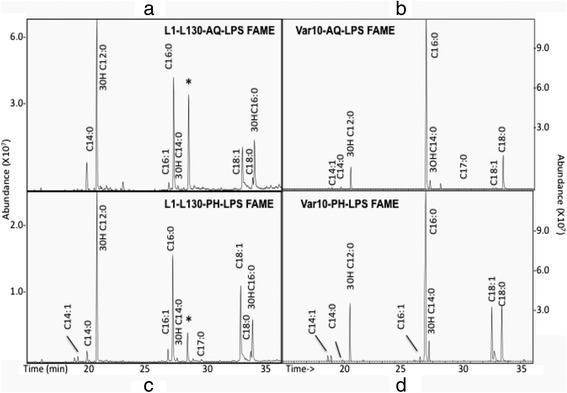
Fatty acid composition analysis of lipopolysaccharide (LPS) from pathogenic and intermediately pathogenic *Leptospira* species. LPS was extracted from the pathogenic species *Leptospira interrogans* serovar Copenhageni strain Fiocruz L1-130 and the intermediately pathogenic species *Leptospira licerasiae* serovar Varillal strain VAR010. LPS fractions were analyzed by gas chromatography–mass spectrometry (GC-MS) as fatty acid methyl ester (FAME) and trimethyl silyl ether-FAME derivatives of hydroxyl fatty acids: (**a**) Copenhageni aqueous layer LPS; (**b**) VAR010 aqueous layer LPS; (**c**) Copenhageni phenol layer LPS; (**d**) VAR010 phenol layer LPS

The fatty acid composition of different LPS fractions from *L. interrogans* serovar Copenhageni strain Fiocruz L1-130 and *L. licerasiae* serovar Varillal strain VAR010 (VAR010) are summarized in Table 2 and their corresponding GC-MS spectra are shown in Fig. 3. The LPSs consist of hydroxyl-lauric acid (3-OH C12:0); palmitic acid (C16:0); stearic acid (C18:0); oleic acid (C18: 1); myristic (C14:0) acid; hydroxyl-myristic acid (3-OH C14:0), hydroxyl palmitic acid (3-OH C16:0), and several other minor fatty acids such as myristoleic acid (C14: 1) and palmitoleic acid (C16: 1). Hydroxylauric (3-OH-C12:0) was present in higher amounts in serovar Copenhageni LPS as compared to that of Varillal, and hydroxypalmitate was only detected in serovar Copenhageni.

**Table 2:** Percentage of fatty acid composition analysis of LPS of pathogenic versus intermediate pathogenic *Leptospira* strains

Fatty acid	Pathogenic L1-130			Intermediate pathogenic VAR010		
	AQ LPS	PH LPS	Ave (± SD)	AQ LPS	PH LPS	Ave (±SD)
Myristoleic (C_14:1_)	0.0	1.32	0.66 (1.98)	0.71	1.37	1.04 (0.33)
Myristic acid (C_14:0_)	0.19	0.60	0.395 (0.205)	0.11	0.38	0.245 (0.135)
Hydroxylauric (3-OH-C_12:0_)	40.02	36.12	38.07 (1.95)	8.24	11.88	10.06 (1.82)
Palmitoleic (C_16:1_)	2.32	3.45	2.89 (0.565)	0.0	1.48	0.74 (0.74)
Palmitic (C_16:0_)	28.67	24.39	26.53 (2.14)	71.34	51.80	61.57 (9.77)
3-Hydroxymyristate (3-OH-C_14:0_)	0.97	0.94	0.955 (0.015)	3.55	4.28	3.915 (0.365)
Margaric acid (C_17:0_)	0.0	0.46	0.23 (0.23)	0	0.24	0.12 (0.12)
Oleic (C_18:1_)	11.78	19.19	15.49 (3.705)	1.37	16.27	8.82 (7.45)
Steric (C_18:0_)	2.57	2.40	2.49 (0.085)	14.47	12.28	13.375 (1.095)
3-hydroxypalmitate (3-OH-C_16:0_)	13.48	11.14	12.31 (1.17)	0.0	0.0	0.0

LPS from *Leptospira interogans* serovar Copenhageni strain L1-130 (L1-130) and intermediate pathogenic, *Leptospira licerasiae* serovar Varillal strain VAR010 (VAR010) were purified from both aqueous (AQ) and phenol layer (*PH*) and subjected for composition analysis separately. Fatty acids in both aqueous (AQ) and phenol (PH) layer LPS extracts were measured and given in the table. Average (*Ave*) and Standard deviation (± SD) values are also presented in this table

### Analysis of KDO

**Fig. 4:**
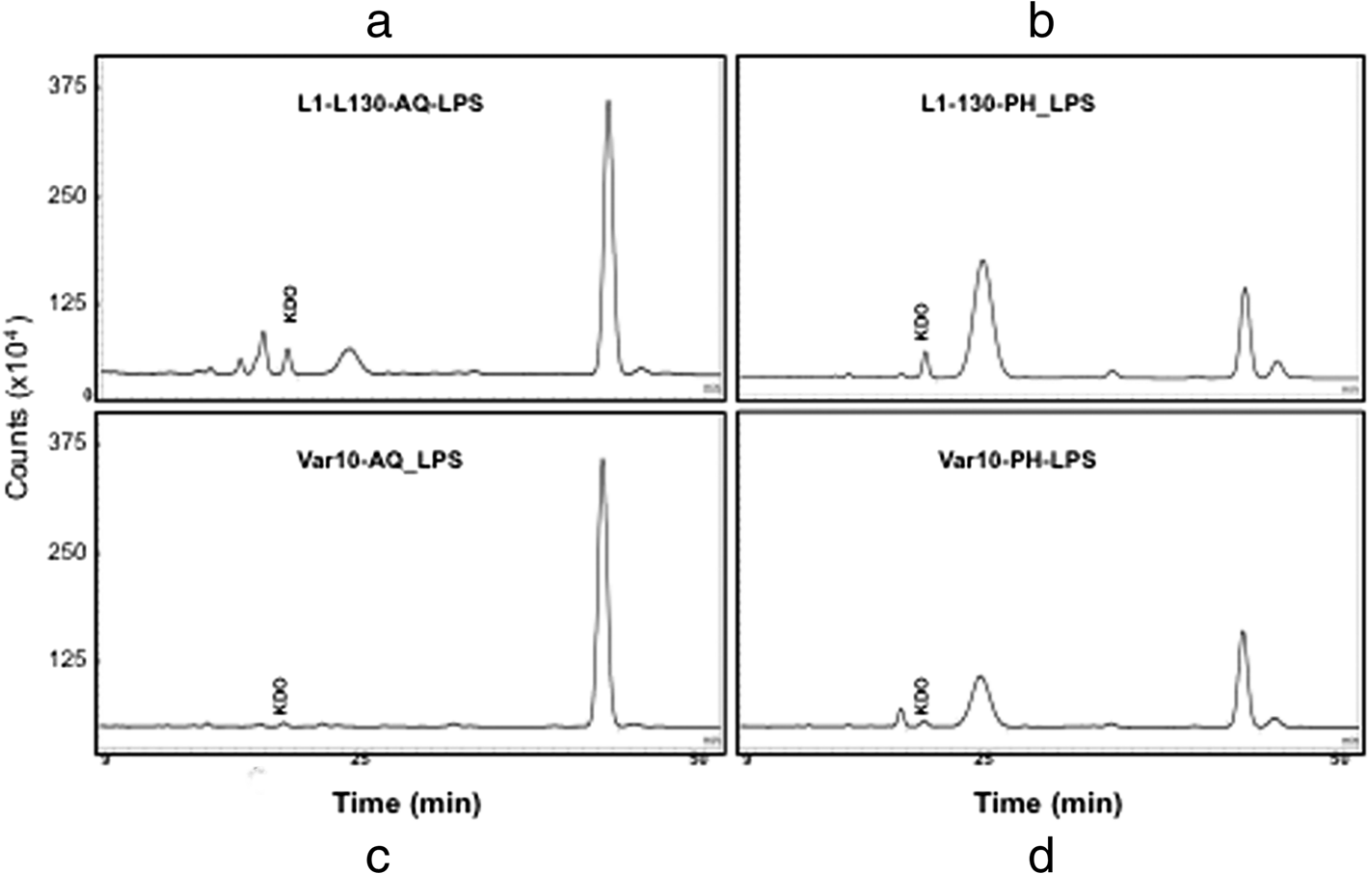
Detection and quantitative estimation of 2-keto-3-deoxyoctonate (KDO) in pathogenic and intermediately pathogenic *Leptospira* species. LPS extracted from the pathogenic species *Leptospira interrogans* serovar Copenhageni strain Fiocruz L1-130, and the intermediately pathogenic species *Leptospira licerasiae* serovar Varillal strain VAR010 was subjected to KDO analysis using reverse phase HPLC with a C18 column after tagging with a fluorophore 4, 5 methylenedioxy-1, 2-phenylenediamine dihydrochloride (DMB). **a** Copenhageni aqueous layer LPS; (**b**) VAR010 aqueous layer LPS; (**c**) Copenhageni phenol layer LPS; (**d**) VAR010 phenol layer LPS

KDO in AQ and PH layer of *L. interrogans* serovar Copenhageni strain Fiocruz L1-130 was estimated to be 3.6 and 4.7 pmole per 12.5 *μ*g of LPS, respectively (Fig. 4a, b). The KDO contents were strikingly low in *L. licerasiae* serovar Varillal strain VAR010 in both phenol and aqueous layer LPS samples (Fig. 4c and d).

### Ultra-structure of Leptospiral LPS

**Fig. 5:**
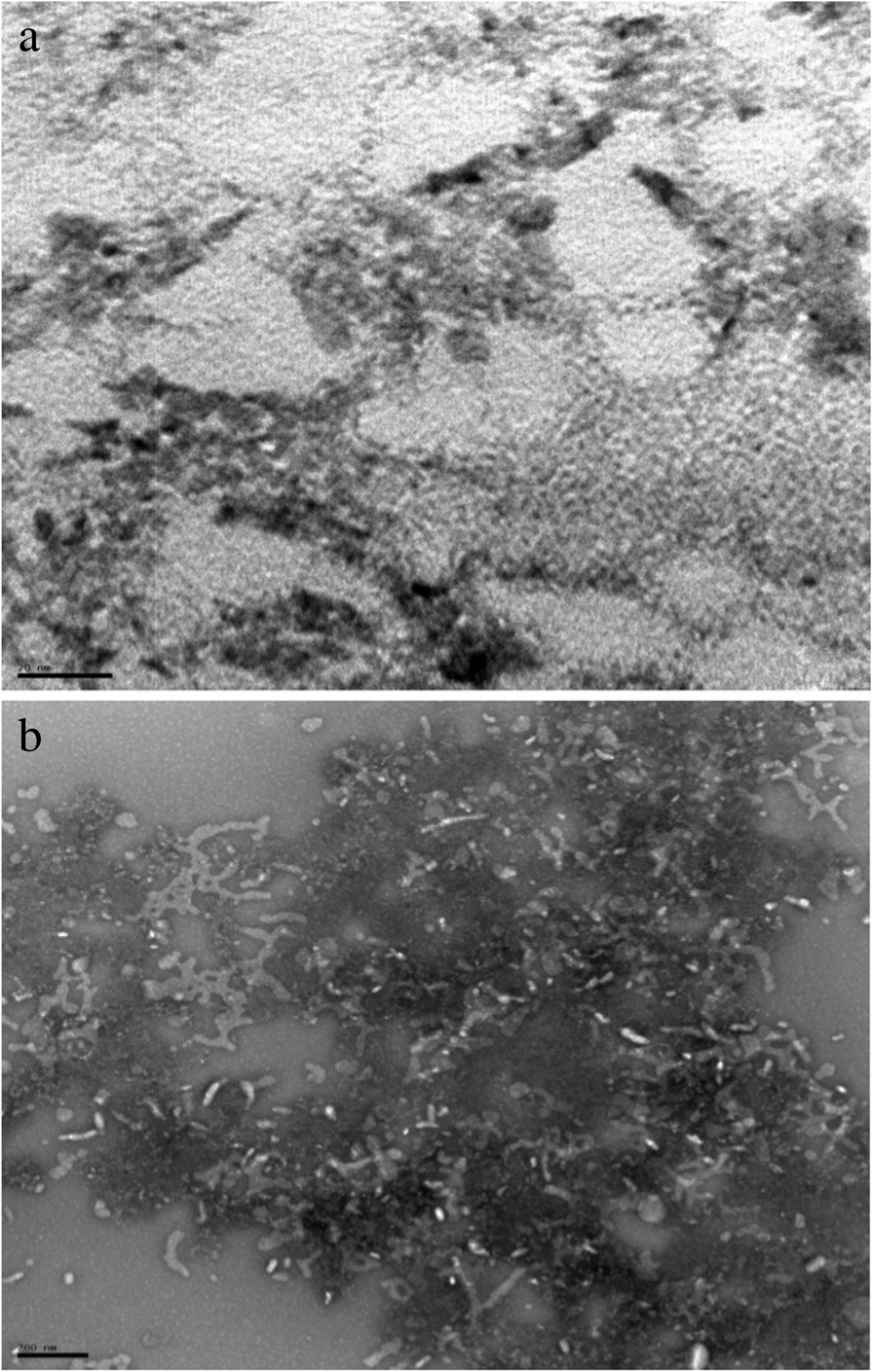
Electron micrograph of lipopolysaccharide extracted from pathogenic and intermediate pathogenic *Leptospira* strains. LPS were spotted on a carbon-coated mesh, air-dried, negatively stained with 2 % uranyl acetate and examined under a transmission electron microscope. The pictures show the different LPS structures of intermediately pathogenic (*L. licerasiae* serovar Varillal strain VAR010, top figure **a** scale bar 0.5 μM.) and pathogenic (*L interogans* serovar Copenhageni strain Fiocruz L-130, bottom figure **b** scale bar 0.2) strains, respectively

Electron microscopic examination of negatively stained LPS extracted from *L. interrogans* serovar Copenhageni strain Fiocruz L1-130 and *L. licerasiae* serovar Varillal strain VAR010 had different morphologies (Fig. 5). VAR010 LPS is a closely packed sheet comprised of interwoven smaller thread-like filaments (Fig. 5a). By contrast, L1-130 LPS consisted of filaments are larger in size, clustered together that did not form any definite sheet like structure (Fig. 5b).

## Discussion

Here we report key experimentally-determined biochemical details of the composition of the lipopolysaccharide (LPS) of *L. licerasiae* serovar Varillal, an intermediate pathogen leptospire with unique antigenicity [18], broad reactivity among people resident in the Peruvian Amazon [18], and an unusually small *rfb* locus comprised of only 6 genes [3]. This analysis of *L. licerasiae* serovar Varillal LPS demonstrated important differences when compared to the LPS of the highly pathogenic *L. interrogans* serovar Copenhageni. We found that *L. licerasiae* Varillal and *L. interrogans* Copenhageni LPS differ significantly in carbohydrate and fatty-acid compositions, particularly in that *L. interrogans* serovar Copenhageni was found to contain fucose, N-acetylglucosamine and N-acetylgalactosamine, FucNAc and QuicNAc and hydroxypalmitate (3-OH-C16:0) while *L. licerasiae* serovar Varillal did not.

While leptospiral LPS does not have potent endotoxigenic properties [25], it has long been known to be the antigen that defines serovar and serogroups, and anti-leptospiral LPS antibodies are responsible for serovar-specific immunity [26–36]. *Leptospira* LPS plays a major role in immunity [37, 38], potential candidate for vaccine development [17] and also anti-LPS monoclonal antibodies used for diagnosis [14, 17]. In contrast, the other human-infecting spirochetes—*Borrelia* spp. and *Treponema* spp.—do not have LPS or an outer membrane [12], so that these biochemical components and structures do not play a role in the biology of these pathogens. Transposon mutants, deficient in LPS production, demonstrated that LPS is a critical virulence determinant and is necessary for colonization of the vertebrate host [12, 37], similar to that of *Salmonella* spp. LPS [39]. However*,* detailed analysis of leptospiral LPS remains understudied, and the present report provides a new approach to such studies.

In our observations, purification of LPS from *Leptospira* species differed from other Gram-negative organisms, in which LPS precipitation primarily occurs from the aqueous phase of hot phenol-water extracts. In earlier studies, aqueous phase *Leptospira* LPS were used for composition analysis, immunological and biological studies [18, 26–28]. However, a substantial amount of *Leptospira* LPS also separates into the phenol layer during LPS extraction procedures like Gram-negative bacteria that contain smooth LPS [9]. Our results indicate that *Leptospira* LPS is distributed differently in phenol and aqueous layer in pathogenic (L1-130) and intermediate pathogenic strain (VAR010). The distribution of LPS in organic and aqueous layers mainly depends on the ratio of hydrophilic saccharide portion and hydrophobic lipid part of the LPS [40, 41]. We analyzed LPS isolated from both aqueous and phenol layers separately to compare chemical composition of serovars Copenhageni and Varillal. The growth temperature of an organism may also influence the LPS composition and solubility. For example, in *Yersinia pestis* the LPS compositions changes under two different growth conditions (mammalian, 37 °C and environmental 25 °C) mimicking host body temperatures [42] and similarly evident in other pathogens [43, 44]. O-antigen content of *L. interrogans* has been observed to change between acute and chronic infection, and when cultivated under *in vitro* conditions [13]. Interestingly, transmission of leptospirosis requires that *Leptospira* survive and adapt to a wide variety of conditions (renal tubules, urine, river, mud, soil, etc.) and characteristics of these adaptations are not clear [13]. We cultured both *Leptospira* species (L1-130 and VAR010) at 30 °C in a medium rich in long chain fatty acids (EMJH medium), a standard method. A potential and important limitation of the present study is that only the LPS of *in vitro* cultivated bacteria were studied. We obtained sufficient amount of LPS from the *in-vitro* cultured *Leptospira* strains (L1-130 and VAR010) for chemical analysis*,* which is currently not feasible from in vivo growing *Leptospira*.

Analysis of LPS by SDS-PAGE followed by periodic acid-silver staining has been used to characterize LPS heterogeneity in different *Leptospira* serovars [27] and other Gram-negative bacteria [9, 33, 34]. The periodic-silver stain gel (Fig. 1) shows that in the L1-130, the fastest migrating LPS component is probably the lipid-A core, and the slower migrating components are O-antigens as seen in other Gram-negative organisms. In VAR010 LPS are very compact (ranges up to 25 kDa) in both aqueous/phenol layers LPS extraction. *E. coli* LPS is characterized by a close succession of regularly spaced bands representing increasing number of O-antigen units. However, in L1-130 the O-side chain patterns may be irregular resulting in a smear-like pattern. The results make evident that the intermediate strain appears to possess the rough type of LPS compared to pathogenic strain that has both smooth and rough form, and further suggests that the O-antigen carbohydrates in *Leptospira* are not simple repeating units. This finding is particularly important as the O-antigen changes have been previously observed in the same pathogenic strain in acute versus chronic *L. interrogans* infection animal model [13] and serial passaging in immuno-incompetent hosts in other organisms [36]. Because of the current lack of a small animal model for *L. licerasiae* (or other intermediately pathogenic *Leptospira*), the VAR010 strain was maintained by *in-vitro* culture, which possibly could have led to some sort of change in LPS molecule. However, anti-VAR010 antisera continue to agglutinate the *in vitro* passaged strain suggesting that the essential antigen determinants are preserved. Our recent data showed that we could infect *Rattus norvegicus* with VAR010 to infect and establish chronic renal infection (unpublished data, Carla Fernandez, Michael M. Matthias and Joseph M. Vinetz, 2015).

We demonstrated that the sugar composition of LPS of L1-130 and VAR010 differs, and that the LPS purified from aqueous and phenol layers of these *Leptospira* is different. Interestingly, fucose, N-acetylglucosamine and N-acetylgalactosamine are only found in L1-130 and not detected in the VAR010 LPS samples. FucNAc and QuicNAc are only found in the aqueous layer LPS from L1-130, and both the sugars are reported to be present on the O-antigen repeat unit of semi-rough or smooth type LPS in different pathogenic bacterial species such as *Vibrio cholera* and *Pseudomonas* [45, 46]. Earlier publications show that there are high content of rhamnose and arabinose in the LPS of pathogenic *Leptospira* species, *L. interrogans* serovar Copenhageni and *L.interrogans* serovar Hardjo [21, 47]. However, we observed in the intermediate-pathogenic *Leptospira* species, VAR010, arabinose amount was two-fold higher compared to rhamnose. Therefore, we hypothesize that arabinose could play a protective role in survival under certain environmental conditions. *Azospirillums* showed that a high arabinose content in exopolysaccharide (EPS) plays an important role in cell aggregation that allows survival in a hostile environment [48, 49].

In Gram-negative organisms lipid A is responsible for the endotixic activity [8]. The lipid A of *L. interrogans* has been reported to be structurally different from other enterobacterial lipid A [25], and to be recognized by the mouse innate immune system by TLR-2 and not TLR-4 [50]. The lipid A center is an essential component for the viability of most bacteria, with a few exceptions. For example, *Neisseria meningitidis* can survive without lipid A in the presence of capsular polysaccharide [51, 52]. The biological activity of LPS is dependent on several structural features of the lipid A. Among them, the presence of phosphate group, substitution of the phosphate residues (with phosphoethanolamine, phosphocholine and methyl groups), alteration of fatty acyl residues, fatty acid type and chain length [53].

The fatty acid moieties present in the lipid A consist of chain lengths between C10-C14 in majority of organisms and few organisms show C16-C18 [8]. For examples, *P. aeruginosa* expresses fatty acid of shorter chain length C10 and C12; *H. pylori* possess longer chain of fatty acids (C16-C18) and *H. influenzae* present C14 fatty acids [54]. In *Enterobacteriaceae* LPS, 3-OH-C14:0 is the dominant 3-OH-fatty acids [55]. The LPS of *E. coli* contains fatty acids with chain length of C12 and 3-OH C14:0 [8]. We analyzed the fatty acid from *E. coli* 011:B4 (Sigma, # L 2630, Data not shown here) indicated the presence of 3-OH C14:0 as the major component. The LPS of VAR010 shows the presence of 3-OH C14:0, however it’s percentage is lot less in pathogenic strains. Interestingly, 3-OH fatty acids C16:0 is only present in L1-130 and not detected in VAR010. In Gram-negative organisms, 3-Hydroxy long-chain fatty acids are essential to the endotoxin activity and also used as a chemical markers of LPS [56], hence the role of 3-OH C16:0 in L1-130 need further evaluations. Hydroxylauric acid present in both VAR010 and L1-130 in different ratio, these finding was reported previously for L1-130 [25]. The fatty acid composition differed between the pathogenic (L1-130) and intermediate pathogenic *Leptospira* (VAR010) that we studied here; for example, fatty acid hydroxypalmitate (3-OH-C16:0) was unique to the pathogenic strain. The *Leptospira* have a unique metabolism that requires a fatty acid-enriched medium [57, 58], and in the host infectious *Leptospira* colonize kidney as a key component of their life cycle [59]. These organs offer a large amount of lipid supply to meet the essential fatty acids requirements for spirochetes growth and multiplication [60]. The fatty acid metabolic pathway of unique fatty acids of pathogenic *Leptospira* LPS can be a good target for the intervention to prevent disease progression. Fatty acid composition has previously been suggested to be related to pathogenicity, and this feature merits for further investigation [61].

We carried out extensive processing to remove the nucleic acids and protein contaminant from the LPS preparation by nucleases treatment, proteolytic digestion, ultracentrifugation and dialysis as per published method of *Leptospira* LPS purification [24]. However, it is possible that glycoproteins that are tightly complexed with LPS and not amenable to protease digestion may contribute to the lipid content. We acknowledge this as a potential limitation of the LPS preparation by available method. The percentages of fatty acids commonly found in both strains such as palmitic acid, stearic and oleic acid are contributions from some non-LPS components such as glycolipoproteins that are co-extracted with the LPS and could not be removed.

KDO (2-keto-3-deoxymanno-octulosonic acid) is an eight carbon acidic sugar found in the LPS of Gram-negative bacteria and it connects the Lipid A part to the O-antigen. The conflicting ability to detect KDO and detection of KDO in some pathogenic strains [11, 18, 26] [24] makes it interesting to understand how sugars might play a substitute role in the absence or limited amount of KDO. We also found KDO in the phenol phase extract of both virulent and intermediate strain and the aqueous phase of the virulent strain only. The amount of KDO in the intermediate was very low with a small peak eluted at the same retention time corresponding to standard KDO and L1-130 KDO peak. It is possible that in the intermediately pathogenic strain other sugars or other form of KDO might also be playing a role in maintaining the weak linkage between O-antigenic carbohydrate and the lipid-A part of LPS. For example, KDO is replaced by 2-keto-D-glycero-D-talo-octonic (KO) in *Burkholderia cepacia,* a Gram-negative bacterial pathogens that causes respiratory infection in cystic fibrosis patients [8].

## Conclusions

This study is the first detailed biochemical composition analysis of an intermediately pathogenic *Leptospira* LPS, and the first to compare leptospiral LPS in *Leptospira* pathogen vs. intermediate species. Many sugars and fatty acid were found in *L. interrogans* Copenhageni L1-130 but not in the intermediately pathogenic *L. licerasiae* Varillal VAR010, which merits further investigation. Current and forthcoming comparative whole *Leptospira* genomic analysis will allow for detailed genetic/genomic, functional and structural analysis of leptospiral LPS, a molecule essential to diverse aspects of this spirochete’s biology, which will improve our understand of mechanisms of pathogenesis and potential vaccine-related interventions.

## Abbreviations

LPS: Lipopolysaccharides
GlcNAc: N-acetyl glucosamine
HexNAc: N-acetylhexoseamine
SDS-PAGE: Sodium dodecyl polyacrylamide gel electrophoresis
GC-MS: Gas chromatography–mass spectrometry
*rfb*: LPS biosynthetic locus
AAALAC: Association for Assessment and Accreditation of Laboratory Animal Care
EMJH: Ellinghausen-McCullough-Johnson-Harris medium
FAME: Fatty acid methyl ester (FAME)
(Tri-Sil)-FAME: Trimethyl silyl ether
HOAc: Acetic acid
AQ layer: Aqueous layer
PH layer: Phenol layer
Ara: Arabinose (Ara)
Rha: Rhamnose
Xyl: Xylose
Man: Mannose
Gal: Galactose
GalNAc: N-acetyl galactosamine
QuiNAc: N-acetyl quinovosamine
FucNAc: N-acetyl fucosamine
KDO: 2-keto-3-deoxymanno-octulosonic acid
KO: 2-keto-D-glycero-D-talo-octonic
TLR: Toll-like receptor
